# Ultrasound-guided percutaneous compartment release: a novel technique, proof of concept, and clinical relevance

**DOI:** 10.1007/s00256-018-3134-y

**Published:** 2018-12-20

**Authors:** Joseph Davies, Valerie Fallon, Jimmy Kyaw Tun

**Affiliations:** 1 Royal London Hospital, Bart’s Health NHS Trust, London, E1 1BB UK; 2 Queen Mary University, London, E1 4NS UK

**Keywords:** Ultrasound, Compartment syndrome, Minimally invasive, Thread release

## Abstract

**Objective:**

Ultrasound-guided thread release (USGTR) is a minimally invasive technique with excellent clinical outcomes currently used in clinical practice to divide the transverse carpal ligament in carpal tunnel syndrome. The purpose of this study is to determine whether this technique can be modified for use in large anatomical compartments in soft embalmed cadaveric models.

**Materials and methods:**

Two operators adapted the USGTR technique for use in muscular compartments of the forearms and legs in a single soft embalmed cadaver. An iterative approach was used to adapt and improve the technique for use in large compartments, using equipment readily available in most radiology departments.

**Results:**

The USGTR technique was successfully modified and both operators were able to accurately divide fascial layers over distances of up to 30 cm using the modified technique. Fascial division was confirmed with ultrasound and dissection.

**Conclusions:**

This adapted technique can successfully be used to divide fascial planes over longer distances than is currently achieved in clinical practice. The improved outcomes associated with USGTR at the carpal tunnel may therefore also be achievable in fasciotomy procedures in larger anatomical compartments. Further study is required to investigate the effects of this modified USGTR technique on intracompartmental pressure.

## Introduction

Numerous anatomical compartments are confined by fibrous layers resulting in low compliance and fixed volume [1]. Processes increasing compartment volume, such as bleeding or edema, increase intracompartmental pressure, potentially compromising neurovascular structures. Most at-risk structures are those passing through fibro-osseous structures such as the carpal, cubital, and tarsal tunnels as well as muscle compartments bounded by fascia.

Conventionally, treatment of compartment syndrome is open surgical fasciotomy, which is associated with complications of open surgery, including pain, scarring, adhesions, and infection [2]. To reduce complications, less-invasive approaches have been developed aiming to maintain efficacy and reduce postoperative pain, improve patient satisfaction, and reduce recovery time vs. open surgery [3, 4]. These include using Metzenbaum or endoscopic scissors via small skin incisions [5, 6] and ultrasound-guided meniscotome instruments [7].

Even less-invasive techniques used in carpal tunnel release (CTR) include endoscopic, [8] ultrasound-guided hook knife [9, 10], and ultrasound-guided thread release (USGTR) [11]. The latter has also been successfully used to divide the first annular pulley of the digits [12] and has been shown to improve outcomes vs. open and endoscopic CTR. USGTR involves using ultrasound guidance to place needles deep and superficial to the structure to be divided. Abrasive thread is passed through the needles, which are then withdrawn, leaving only the thread in place. An oscillatory movement on the thread while traction is applied divides the structure, in a similar manor to a cheese wire.

The USGTR technique has not, to our knowledge, been used in larger compartments. We hypothesize that, due to its truly minimally invasive nature, outcomes for USGTR procedures in large compartments could be improved compared to existing techniques.

**Fig. 1:**
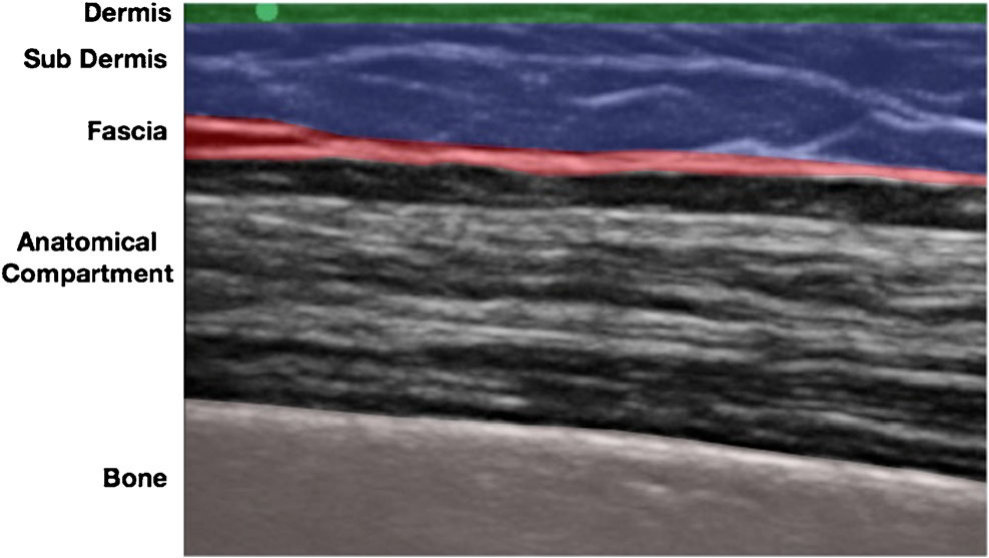
Longitudinal ultrasound image of the extensor compartment of the forearm of a soft embalmed human cadaver demonstrating the ultrasound appearances of key anatomical structures

We speculate that USGTR can be adapted for this purpose because many anatomical compartments prone to compression syndromes are superficial and are therefore easily visualized under ultrasound guidance (Fig. 1). In addition, important local neurovascular structures are readily identified and thus avoided using ultrasound [13]. Before clinical studies can be undertaken, in this paper we have set out to determine the feasibility of applying USGTR to larger anatomical compartments in the limbs of a soft embalmed cadaver.

## Materials and methods

### General design

Two operators with 10 years of combined experience performing ultrasound-guided procedures each performed USGTR of the posterior and peroneal compartments in the leg and the antebrachial fascia of the forearm in a single soft embalmed cadaver. An iterative approach to performing the procedure with incremental improvements in technique was made with a variety of instruments. Both upper and lower limbs were used to refine the technique. The primary outcome was to determine if fascial layers could be divided in the upper and lower limbs of the cadaveric model. Ultrasound assessment of the fascial layer was performed after each procedure to assess whether the extent of fascial division could be determined. The true extent of fascial division and assessment of damage to surrounding structures was then assessed by dissecting the specimen. Secondary outcomes were descriptions of the iterative process and the determination of the optimum equipment and potential problems and pitfalls associated with various techniques and with the cadaveric model itself. Technical parameters including the maximum length of fascial division and procedure time were also evaluated. Procedures were conducted in a licensed dissection facility. Prior to the study, the cadaveric specimen was free of visible signs of limb trauma.

### Human cadaver

The study was conducted using soft-fix human cadavers, as this has been shown to provide a model which more closely resembles the mechanical and ultrasound properties of in vivo human tissue [14]. The soft-fix embalming process also allows for simulation of human disease such as limb compartment syndrome [15] and so initial data collected in this study will be directly comparable to any future work involving compartment pressure monitoring.

### Ultrasound

Sonosite Edge II medical-grade ultrasound machine with a 13-6 MHz 6-cm linear array ultrasound probe was used.

### Surgical instruments

- Small surgical blade
- Blunt tunneler, such as that used in Hickman ® line insertion (Bard Access systems Inc. Salt Lake City, UT, 84116, USA).
- 2–0 or 0 braided silk suture

## Results

Using an iterative approach to incrementally improve technique, progressively more successful attempts were made to divide superficial fascial planes in the upper and lower limbs of the cadaveric specimen. The maximum length of fascial division was dictated by the length of blunt tunneler, which measured approximately 30 cm in length. Longer fascial releases would be technically feasible but would either require a longer tunneler or repeating the thread release process over adjacent lengths of intact fascia. Once the operators had refined the technique and were familiar with each of the steps, the thread release procedure could be completed in no more than 15 min.

**Fig. 2:**
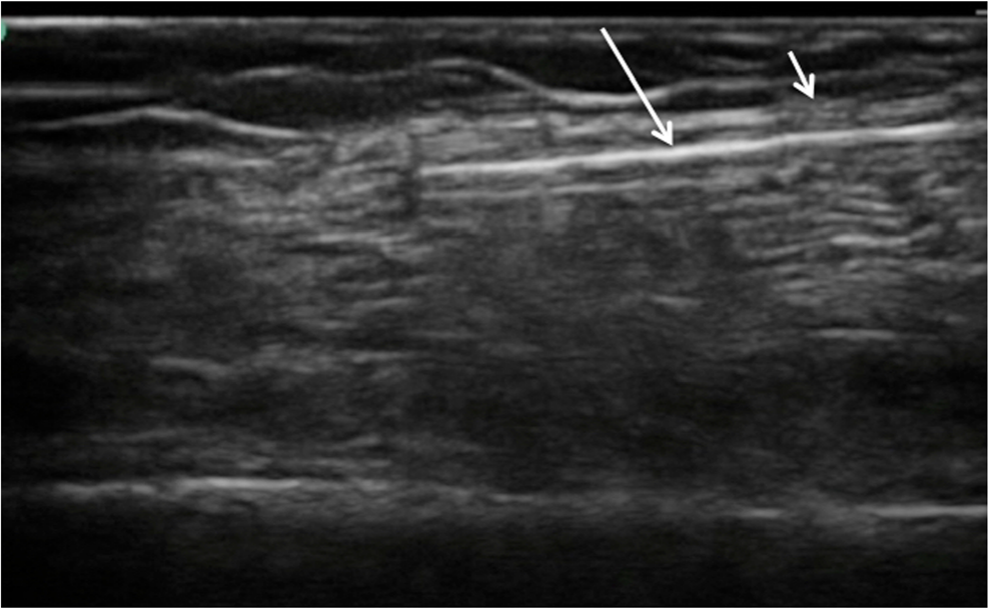
Real-time ultrasound image of blunt tunneling (*long white arrow*) device passing immediately deep to fascial plain (*short white arrow*)

**Fig. 3:**
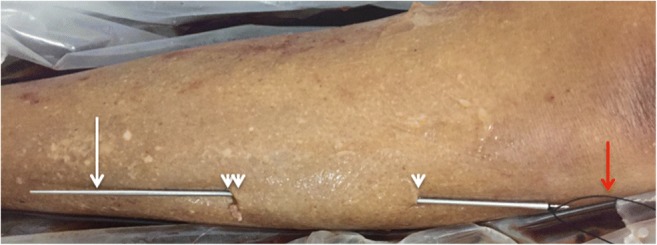
First pass: deep to fascial layer: Tunneling device (*long arrow*) inserted through small proximal incision (*single arrowhead*) and passed deep to the tissue plane to be divided before being retrieved and brought to the skin surface through a small distal incision (*double arrowheads*). Braided suture (*red arrow*) is attached to the tunneler

**Fig. 4:**
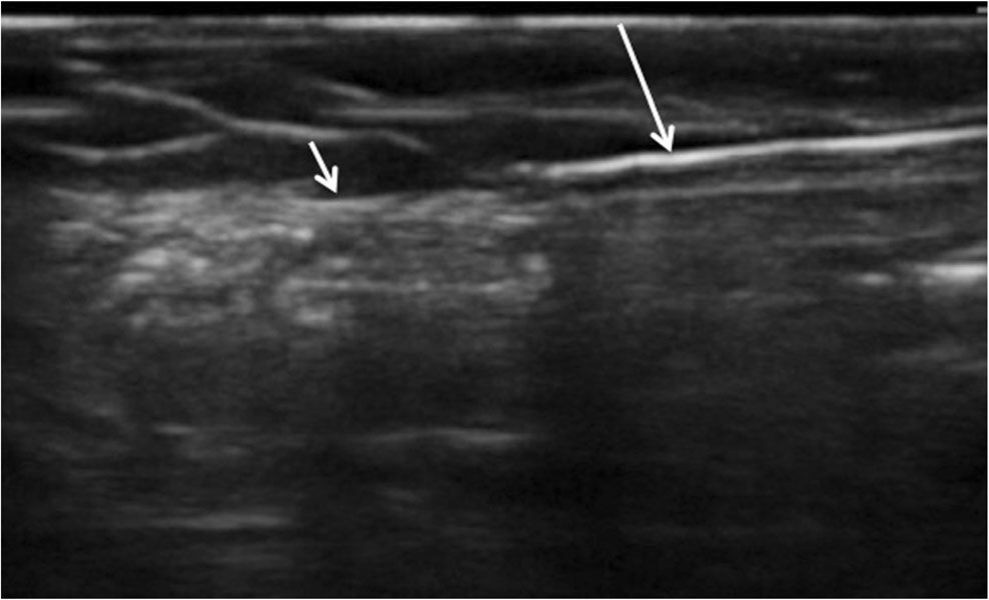
Real-time ultrasound image of blunt tunneling device (*long arrow*) passing immediately superficial to fascial plain (*short arrow*)

**Fig. 5:**
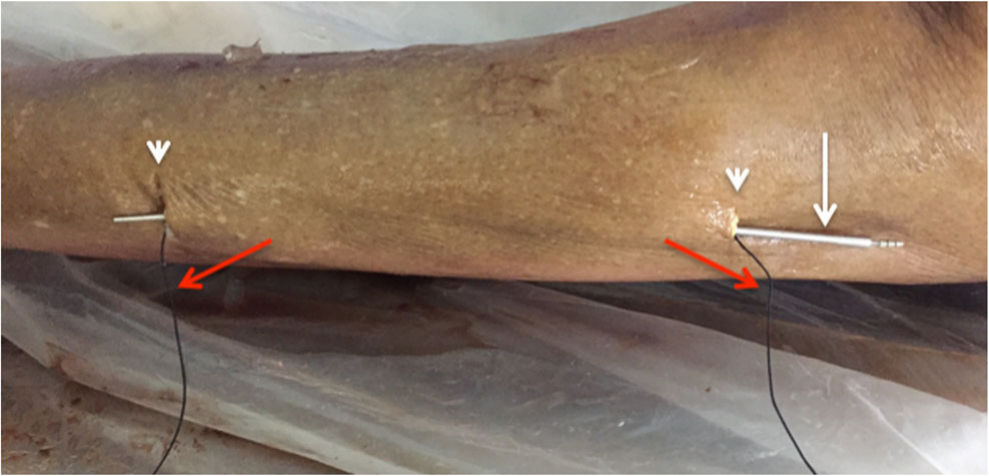
Second pass (superficial to fascial layer): Tunneling device (*white arrow*) passed in the small cutaneous incisions superficial to the tissue plane to be divided. Silk thread is then tied to the tunneler before being pulled through

**Fig. 6:**
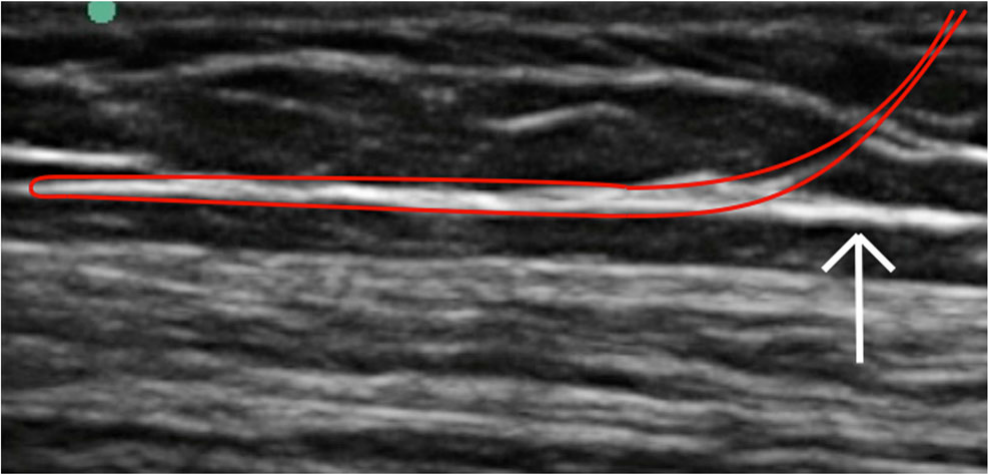
Ultrasound image demonstrating the intended location of the thread (*red line*), looped immediately superficial and deep to the fascial layer to be divided (*white arrow*)

**Fig. 7:**
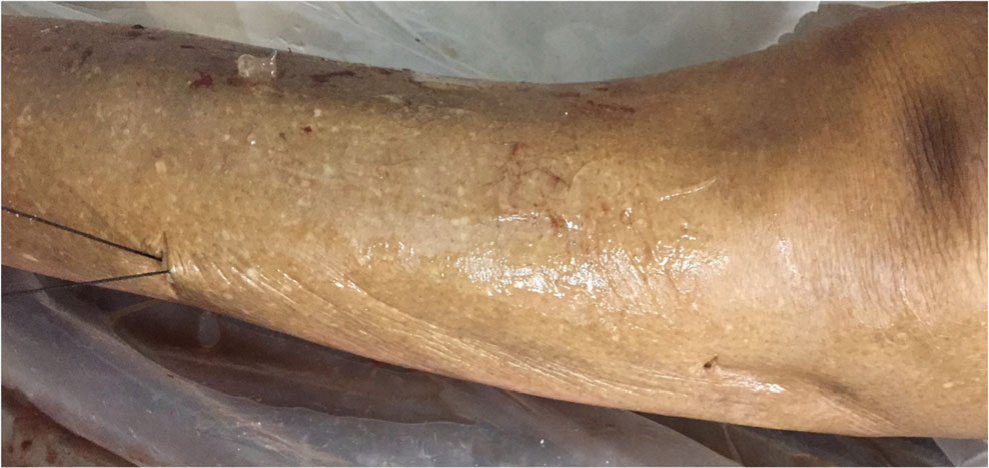
Silk thread is looped around the facial plane to be divided. Firm oscillation of the free ends creates sufficient cutting force to release the compartment

**Fig. 8:**
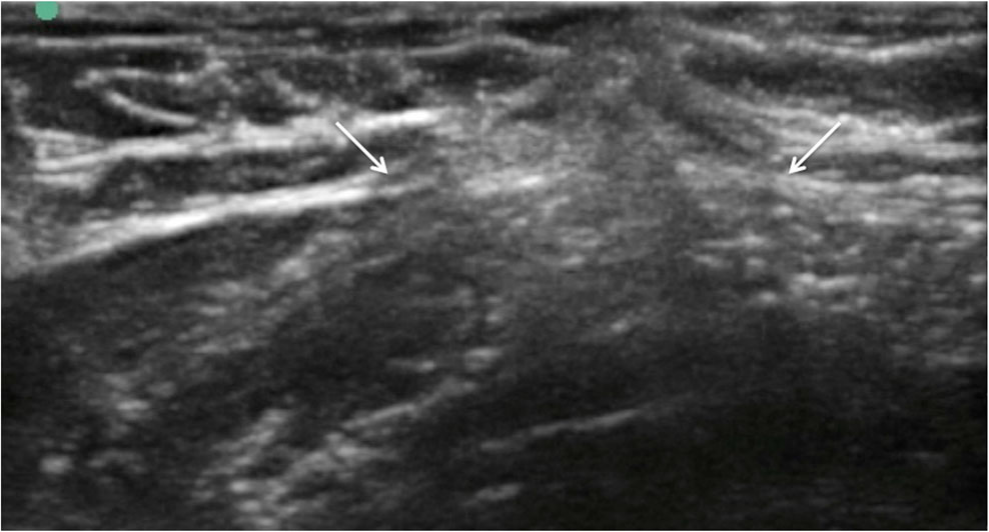
Ultrasound image of fascial layer post thread release demonstrating divided and retracted fascial edges (*white arrows*)

**Fig. 9:**
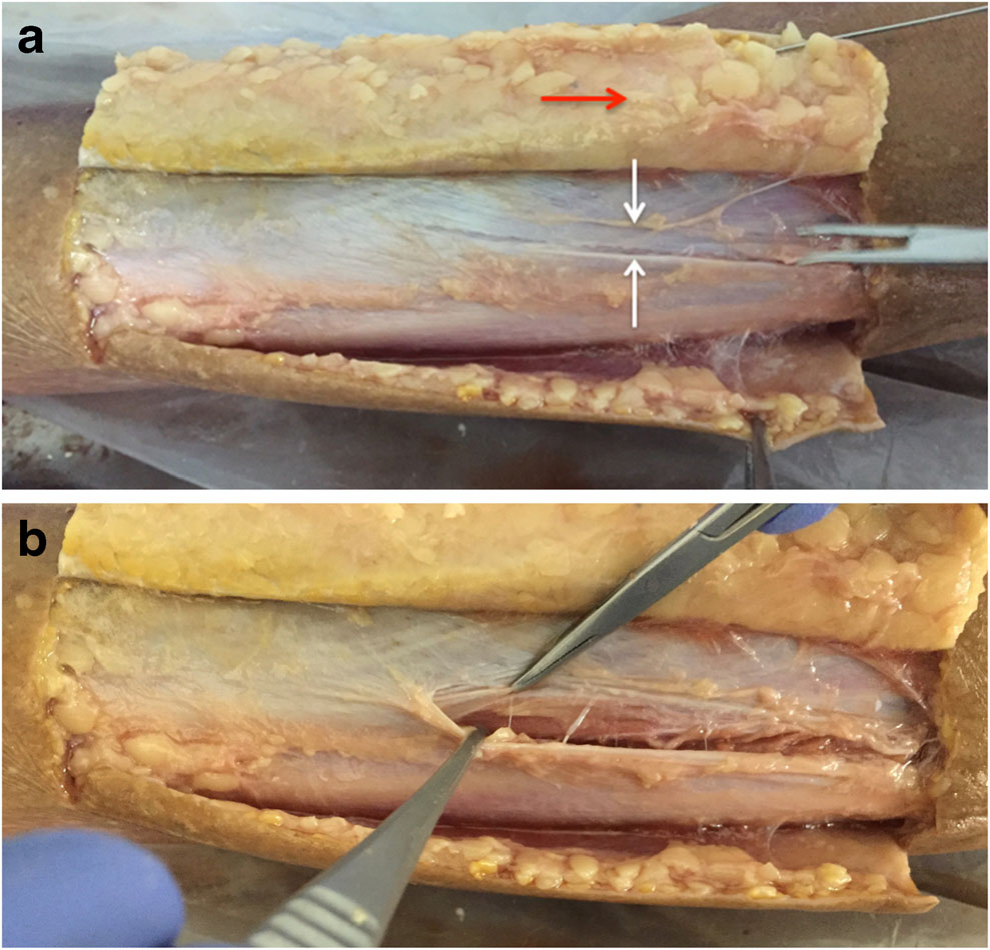
**a** Dissection reveals cleanly divided fascia (*white arrows* delineate free edges of divided fascia). **b** There is no significant trauma evident to the overlying subcutaneous fat (*red arrow*) or the underlying muscle

The most successful technique involved using a small incision to dissect down to the fascial plane, which is then pierced using a small surgical blade, such that a blunt tunneling device can be inserted immediately deep to the tissue plane to be divided. Using real-time ultrasound guidance, the blunt tunneling device was then passed adjacent to the fascia for the required longitudinal distance, keeping the tip visualized throughout (Fig. 2). When the tip of the blunt tunneler has passed the required distance under the tissue plane, a second small incision is made above the tip so that it may be brought to the skin surface (Fig. 3). A 2–0-diameter braided silk suture is then tied to the end of the tunneler before being pulled through, leaving the silk thread deep to the tissue plane. The process is then repeated in the opposite direction with the blunt tunneler passing through the same incisions and passed, again under direct ultrasound guidance, superficial to the tissue plane to be divided (Figs. 4, 5). The silk thread is once again tied to the end of the blunt tunneling device and pulled through so that it now passes immediately deep and superficial to the tissue plane to be divided (Figs. 6, 7). The ends of the suture are then oscillated under tension creating a “cheese wire” effect, thus dividing the fascia. Evidence of fascial division could be obtained using ultrasound (Fig. 8) and was confirmed following open dissection (Fig. 9a, b).

The soft embalmed cadaveric specimen proved to be a high-fidelity model for use with ultrasound. The ultrasound appearance of cutaneous, subcutaneous, fascial, and muscular structures was, subjectively, almost identical to that in live specimens. Adjacent neurovascular structures could be identified in the cadaveric model although with more difficulty than in live models due to lack of Doppler signal. Despite this, post-procedure dissections revealed that no critical structures were divided in any of the fascial divisions performed. Post-procedure ultrasound assessment was also able to accurately determine the extent of fasciotomy.

The critical innovation was the use of a blunt tunneling device to place the thread. Trials using needles of various gauges and stiffnesses were unsuccessful, as all needles were found to be too difficult to steer over long distances due to insufficient needle tip control and the tendency of the needle to pass through the fascial plane, from superficial to deep, rather than remaining closely applied to it. Successful thread placement was achieved using a blunt tunneling device designed for tunneling Hickman ® Lines (Bard Access Systems, Salt Lake City, UT, USA). This device offered sufficient length and could easily be steered under ultrasound guidance due to increased rigidity and also preferentially followed tissue plains as a result of its blunt tip. This device also enabled the thread to be tied to one end and pulled through, rather than needing to be threaded through a needle lumen. A commonly encountered problem with the technique was failure of the thread during division of the fascia. This occurred in approximately 50% of all attempts and resulted in incomplete fascial division.

During the study, an attempt was made to simulate raised intracompartmental pressure by injecting saline. This was unsuccessful, as the fluid was able to cross the fascial plane with ease. Simulating raised intracompartmental pressure could not therefore be achieved using this method.

## Discussion

To our knowledge, this is the first study investigating the feasibility of releasing large fascial compartments using a USGTR technique on a soft embalmed cadaveric model. The USGTR technique previously described by Guo et al. [16] has demonstrated significantly improved clinical outcomes compared to open and endoscopic carpal tunnel release procedures [11]. However, this technique is not easily applied to larger fascial compartments. The modifications to the USGTR technique described in this paper, which principally include the use of a blunt tunneling device to achieve thread placement, enable the fasciotomy procedure to be carried out on long segments of fascia, such as is required to treat compartment syndrome in the forearms or legs. Our results also demonstrate that the modified USGTR procedure can be carried out in a cadaveric specimen in a minimally invasive fashion within a short time frame and without damage to critical neurovascular structures. It is therefore possible to hypothesize that the improved clinical outcomes seen with the USGTR technique at the carpal tunnel may also be possible to achieve in larger fasciotomy procedures in the leg and forearm. The modified USGTR technique described here may therefore be suitable for further study and consideration of clinical use.

Several limitations and areas for further study have been identified. A commonly encountered problem was failure of the silk polyfilament suture used to “cheesewire” the fascial plane. This occurred in approximately 50% of cases, prior to complete division of the fascia. The suture we used was not designed for this purpose and was only 2–0 in diameter. A larger diameter suture with greater tensile strength could be used or a specifically designed cutting thread employed such as that used by Guo et al. [16]. It is also unknown if the physical properties of the soft embalmed fascia closely resemble those of live tissues, and therefore, if our results would be reproducible in live models.

Compartment pressure was not measured in this study, and so the effect on intra-compartmental pressure of fasciotomy could not be measured. However, there is anecdotal evidence from previous studies demonstrating good symptomatic relief from minimally invasive percutaneous fasciotomy [6].

Before this modified USGTR procedure can be considered for clinical use, further work is required to answer these questions and in particular to determine the effect of the USGTR on intracompartmental pressure.
